# Effects of landscape structure on restoration success in tropical premontane forest

**DOI:** 10.1038/s41598-022-16542-3

**Published:** 2022-08-04

**Authors:** Miriam San-José, Leland K. Werden, Francis H. Joyce, J. Leighton Reid, Karen D. Holl, Rakan A. Zahawi

**Affiliations:** 1 Lyon Arboretum and School of Life Sciences, University of Hawai’i at Mānoa, Honolulu, HI USA; 2 Charles Darwin Research Station, Charles Darwin Foundation, Av. Charles Darwin, Puerto Ayora, Galápagos Ecuador; 3 Department of Environmental Systems Science, ETH Zürich, Zürich, Switzerland; 4 Plant-for-the-Planet Foundation, Munich, Germany; 5 Environmental Studies Department, University of California, Santa Cruz, CA USA; 6 School of Plant and Environmental Sciences, Virginia Tech, Blacksburg, VA USA

**Keywords:** Environmental sciences, Restoration ecology

## Abstract

Reversing large-scale habitat degradation and deforestation goes beyond what can be achieved by site-level ecological restoration and a landscape ecology perspective is fundamental. Here we assess the relative importance of tree cover and its configuration on forest-dependent birds and late-successional tree seedlings in restoration sites in southern Costa Rica. The abundance and species richness of birds increased in landscapes with more corridors, higher tree cover, and lower levels of fragmentation, highlighting the importance of riparian corridors for connectivity, and continuous tree cover as suitable habitat. Landscape variables affected abundance and species richness of seedlings similarly, but effects were weaker, possibly because seedlings face establishment limitation in addition to dispersal limitation. Moreover, the scale of landscape effects on seedlings was small, likely because proximal individual trees can significantly influence recruitment in restoration plots. Results underscore the importance of incorporating landscape-level metrics to restoration projects, as knowing the extent, and how the landscape may affect restoration outcomes can help to infer what kind of species will arrive to restoration plots.

## Introduction

We are in the UN-declared Decade on Ecosystem Restoration (2021–2030) and many nations have committed to ambitious initiatives to increase forested areas in their countries (e.g., Bonn Challenge, Initiative 20 × 20, One Trillion Trees)^1^. However, reversing large-scale degradation and deforestation, necessitates approaches beyond what can be achieved by site-level ecological restoration^2^. As such, a landscape ecology perspective is fundamental to achieving ambitious forest restoration goals, particularly as restoration needs to be implemented at scale. Filling the knowledge gaps of how landscape structure affects biodiversity is essential to improve large-scale ecological restoration outcomes. Landscape structure studies can also help prioritize areas for restoration, design protected area networks^3,4^, and assess the integrity of multifunctional landscapes^5,6^.

Landscape structure refers to the composition (i.e., extent of different land covers) and configuration (i.e., spatial arrangement of these land covers) in a determined area^7^. Undoubtedly, forest cover is the most important factor promoting tropical forest biodiversity and driving recovery^8,9^, and, typically, some landscape structure compositions (e.g., greater extent of forested areas) and configurations (e.g., high forest connectivity) are more biodiversity-friendly than others^5^. However, the configuration of forest cover is critical at intermediate (20–50%) levels in the landscape, when the values distribution is highly variable^7,10^. For example, the number of forest patches peaks in abundance and variability at ~ 20% forest cover^10^. This may affect ecological restoration outcomes, but to our knowledge, there are no studies comparing how different forest configurations at the landscape scale affect the recovery of different taxa within restoration plantation plots.

The study of landscape connectivity has great potential to improve restoration planning and outcomes. Structural landscape connectivity (hereafter ‘connectivity’) corresponds to the spatial relationships between the structural elements of the landscape (e.g., forest fragments) and is independent of the ecological characteristics of the focal species^11,12^. Understanding landscape connectivity is particularly useful in restoration actions to identify areas where the implementation of new connector elements (e.g., live-fences, isolated trees, fragments) could optimize connectivity in fragmented landscapes^13^. To bridge the gap between landscape connectivity models and actual restoration actions, we need to incorporate spatial modeling into the restoration planning process^13,14^.

Landscape structure can also drive forest restoration outcomes^15^. In other words, the position and immediate habitat surrounding a restoration plot affect restoration outcomes, since landscape configuration is related to seed disperser behavior and to the presence of seed sources. For example, seed-dispersing birds can adjust their behavior as a response to spatial modification in resource distribution and landscape connectivity. Birds fly longer distances and visit more forest patches and remnant trees as forest cover decreases, and increasing forest isolation results in longer flight distances and increases the use of trees as stepping-stones^16^. In turn, plant species richness recovers faster in sites surrounded by higher forest cover^17^, although the effects of surrounding landscape metrics on flora recovery can be weaker than site-level processes^18^.

Restoration experiments at the landscape-scale are rare. However, in southern Costa Rica, K. Holl, R. Zahawi, R. Cole and collaborators established and monitored restoration plots spread across a ~ 100 km^2^ area on degraded farmlands (mostly pastures) starting in 2004^19^. This restoration experiment consisted of three treatments: natural regeneration, planting clusters of trees (applied nucleation) and planting the whole area with trees (plantation). For simplicity of interpretation, and given different spatial distribution of replicate plots within each site, as well as fewer replicates of other treatments, we only used the plantation plots in this study. The more homogeneous vegetation recovery in plantation sites facilitates a more robust assessment of effects of the surrounding landscape on the four response variables in this study. Plantation plots quickly developed a closed canopy and developed a shaded environment with a diverse understory, more similar to reference forests than natural regeneration^18^. Detailed information on biodiversity recovery over time make this experiment suitable for testing the utility of landscape ecology metrics in predicting restoration outcomes. Many papers published from this study suggest that tree cover in the landscape surrounding restoration sites has had a weak effect on tree seedling recruitment within sites^18,20–22^, which might be because the presence and proximity of a single parental tree can drastically increase the abundance of seedlings of that species within a single restoration plot^23^. However, we do not know the relative effect of different tree elements (e.g., live-fences) and landscape structure variables on restoration outcomes. Here, using a multivariate and multi-spatial scale approach, we examined the relative effects of tree cover and its configuration (i.e., degree of fragmentation, remnant tree density, patch aggregation index, mean inter-patch distance, largest patch index) on the abundance and diversity of late-successional tree seedlings and forest-dependent birds across a 7-year timeline of vegetation recovery and bird surveys.

We assessed four response variables (i.e., abundance and species richness of both forest-dependent birds and late-successional seedlings) that are likely to be influenced by tree cover configuration^24^, and indicate whether a forest has recovered a species composition similar to minimally disturbed reference forest. We hypothesized that the effects of fragmentation on biodiversity are more likely to be positive than negative as a higher number of fragments or patches promotes landscape connectivity^25,26^. Hence, high patch density and smaller distances between patches (aggregation of patches) may promote dispersal. Forest-dependent birds are expected to be more sensitive to deforestation as they rely heavily on forest resources for survival (e.g., habitat, diet) so, we expected birds to be strongly and positively affected by the percentage of tree cover. Further, some forest-dependent bird species move through the landscape using corridors, remnant trees, and live-fences, so the number of connectors in the landscape should be positively associated with bird diversity. Whether seedlings recruit in a given site depends on seed sources, animal dispersers (such as birds and mammals), and establishment limitation factors^27,28^. Forest cover represents a rich source of seeds, but so do remnant trees in the landscape, which can greatly contribute to seed sources^23^. As late-successional tree species are mainly found in old-growth forest, we expected a positive effect of tree cover on seedling richness and abundance. Finally, we expected the same direction of the effects of tree cover configuration on seedling recruitment as for birds, given that birds are important seed dispersers in wet tropical forests^29^.

## Methods

### Study site

**Figure 1:**
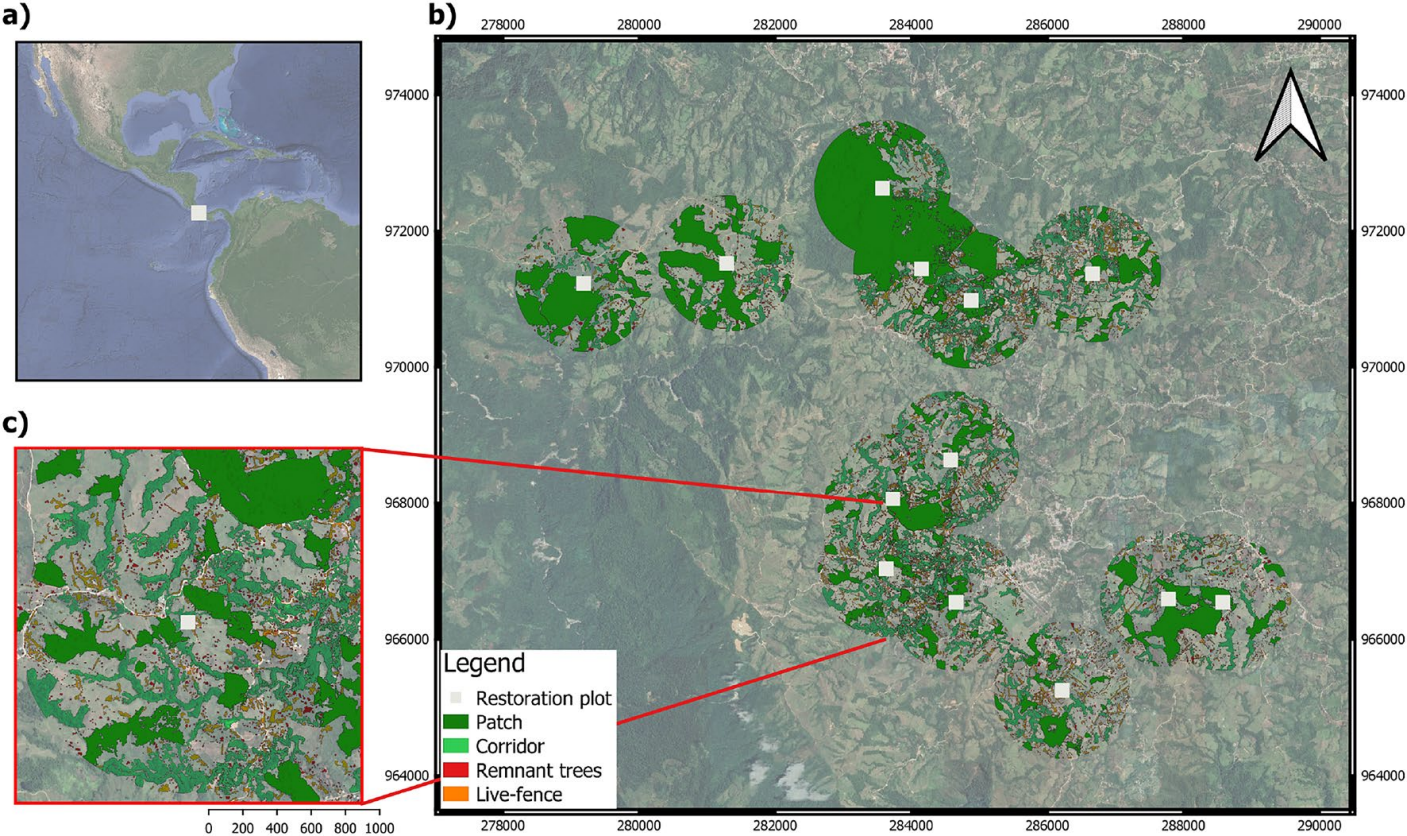
Location of the 13 focal restoration plots (white squares) and their respective surrounding landscape (900-m radius) in southern Costa Rica (**a**, **b**). We show in detail an example of one of these landscapes with its respective classified tree elements (**c**). Map created by MSJ with Quantum GIS v3.10.12 (https://www.qgis.org).

The study region is within the tropical premontane wet forest zone in Coto Brus county, southern Costa Rica (8° 44’–8° 47’ N, 82° 56’–82° 57’ W; Fig. 1). Regionally, these forests are distributed from 600 to 2000 m.a.s.l., are characterized by 2100–4400 mm annual rainfall, with a temperature ranging from 19 to 26 °C, wind-blown mist, and frequent presence of clouds^30^. By the early 1980s, this area had lost ca. 75% of its original vegetation and is at present a mosaic of mixed-use agricultural fields, cattle pastures, and secondary and old-growth forest fragments^31^. Within this matrix, the Las Cruces Biological Station reserve protects ~ 200 ha of old-growth forest and other areas under various stages of recovery (Fig. 1; 365 ha overall). It is one of the largest remaining fragments in the area. Here the landscape is very heterogeneous and agricultural land practices retain some connectivity through riparian corridors, live-fences and isolated trees^21^.

We surveyed a total of 13 sites for seedlings and 12 for birds ranging in edge-to-edge distance among them 800 m–10,500 m (mean = 4555 m, Fig. 1). At each site, in 2004, we established 50 × 50 m (0.25-ha) plantation plots with rows of planted trees across the entire plot using four species: *Terminalia amazonia* (J.F. Gmel.) Exell (Combretaceae), *Vochysia guatemalensis* Donn. Sm. (Vochysiaceae), *Erythrina poeppigiana* Walp. Skeels, and *Inga edulis* Mart. (Fabaceae). The canopy closed before seven years^32^. The whole experiment and restoration treatments are detailed in Cole et al.^20^. For the purposes of this study, we used bird and tree seedling data collected within three years (2011–2017) of the high-resolution aerial imagery from 2014 on which tree cover classifications were determined. We used these years to match with the date of landscape tree cover image, as we assumed that the landscape in the region did not change substantially in that period.

### Bird composition

Each of the 12 plantation plots were surveyed three times annually from 2011 to 2017 by a single observer, Juan Abel Rosales. Each plot at each site was actively searched randomly for 20 min per observation, and all birds detected visiting the plot (by sound or sight) were recorded. All surveys were conducted from sunrise (~ 05:30) until 09:00. Birds flying over plots were excluded from analyses. For detailed information on the field survey methodology see Reid et al.^33^. From all detections recorded, we extracted observations of species categorized as highly forest-dependent by BirdLife International^34^. As defined in Buchanan et al.^34^, these species have high forest-dependence that is characteristic of the interior of undisturbed forest and almost invariably breed within the forest. While they may persist in secondary forest and forest patches if particular ecological requirements are met, they are usually less frequently encountered in such situations. We chose to only include forest-dependent birds in this study because generalist frugivores are known to be less sensitive to landscape variables^35^, and a preliminary analysis including all frugivores weakened explained deviance in all models. We used the cumulative number of species recorded over the seven survey years to determine species richness observed at each plantation plot and the average number of detections per year for each species as a proxy for abundance (hereafter ‘abundance’, see Reid et al.^33^) as our two forest-dependent birds response variables. We then evaluated sample completeness within each site using the estimator of sample coverage proposed by Chao and Shen^36^. This estimator indicates the proportion of the total number of individuals in an assemblage that belongs to the species represented in the sample.

### Tree seedling recruitment

We measured tree seedling recruitment (hereafter seedlings) at four sampling transects located within each plantation (n = 52 transects in total). All tree recruits ≥ 0.2 and < 1 m height were censused within four 1 × 2 m quadrats along 1 × 8 m recruitment sampling belt transects^18,21^. Tree species’ successional stage was determined based on the expert opinion of two taxonomists who have each worked in the region for > 20 years, herbarium specimens at Las Cruces, and literature resources^37–39^. Late-successional species are those mainly found in the interior of old-growth forests; they are generally hard wooded, slow growing, shade tolerant, and long-lived species. During each annual survey, new recruits were permanently tagged, identified to species, and their height measured. For this study, we considered the sum of new recruits for each species in the period 2011–2017, and the total species richness accumulated within that period as seedling response variables.

### Landscape structure

We used a finely detailed tree cover map created by hand digitizing photographs taken in 2014 by commercial QuickBird satellites with 0.61-m spatial resolution in Google Earth for the canton of Coto Brus (for additional details see Mendenhall et al.^40^). Tree cover included the percentage of the landscape covered by old-growth and secondary forest fragments of all sizes, single or groups of remnant trees, live-fences, hedgerows, as well as non-native timber and fruit tree plantations. Tree cover represents any source of fruits and seeds within the landscape^5,41^ and more extensive tree cover indicates larger forest patches, which provide habitat to forest specialist birds. Total tree cover in the landscape surrounding the restoration plots (radius 900 m, ~ 255 ha) ranged from 30 to 79% (Supplementary Fig. 1). Following a combination of several authors’ criteria^5,16,42,43^ and personal field observations, we classified all tree cover elements in the landscapes according to their area (A), perimeter (P), and compactness (SI) where the compactness ratio of the polygons was calculated using the shape index formula SI = P/﻿$2\sqrt{A\pi }$^44^. A criterion to distinguish live-fences, remnant trees, fragments, and corridors was to assign threshold shape index values according to the minimal observed values for well-detected live-fences (n = 45) and corridors (n = 15) within the study region. Categories include: (1) patches—A ≥ 0.25 ha, SI < 2 and wider than 40 m; (2) corridors—A > 0.25 ha and SI > 2; (3) remnant trees—A < 0.25 ha and SI < 1.6; and (4) live-fences – A < 0.25 ha and SI > 1.6. For all landscape classification we used Quantum GIS v3.10.12^45^.

Then, we obtained the percentage of the landscape covered by corridors (riparian and non-riparian) and live-fences as both are important connectivity elements in the landscape, they are widely used by birds and other animals, may promote long-distance dispersal^11,46^, and can also be seed sources for many species^47,48^. Fragmentation was measured as the number of tree patches divided by landscape area (n/ha). This classical fragmentation metric is inversely related to mean patch size^7^. Thus, for a given land cover area, fragmentation increases the edge-to-core ratio at the landscape scale, potentially increasing edge effects (i.e., small-scale process). Yet, as it is also positively related to landscape connectivity and to the number of (sub)populations (seed sources) in the landscape, it can also have stronger effects on seed dispersal over larger spatial scales^25,26^. Remnant tree density was calculated as the number of remnant trees divided by landscape area (n/ha). Small clusters of trees and isolated trees function as stepping stones in the landscape, promoting animal movement, and hence, landscape connectivity^49^. They also constitute seed sources^23^. The patch aggregation index is a percentage of like-adjacencies (nearness of the same land cover) among forest patches, where a single compact patch has a maximum aggregation^50^. Aggregation refers to the degree of clumping of patches, where more aggregated patches facilitate inter-patch movement, promoting dispersal across the landscape^51^. The mean inter-patch distance is the Euclidean or straight-line (edge-to-edge) distance among all forest patches in the landscape and it is a simple measure of patch context and has been used extensively to quantify degree of landscape isolation and act as a proxy for structural connectivity at the landscape scale^52^. Finally, the largest patch index represents the percentage of the landscape comprised by the largest patch which can provide important habitat for forest-dependent birds and late-successional trees. This metric has been identified as a driver of forest restoration success^15^.

We determined landscape metrics for 17 progressively larger circular areas around each plantation plot (radius from plot edge = 20, 40, 60, 80, 100, 150, 200, 250, 300, 350, 400, 450, 500, 600, 700, 800, and 900 m; Supplementary Fig. 1). Larger radii were included following a study indicating that the average distance traveled by toucans with seeds retained in their body is approximately 700 m^53^ while manakins’ longest flight distance in the region was 600 m^54^. As such, our larger landscape size is an ecologically meaningful distance for seed dispersal processes, while also minimizing as much as possible the overlap among landscapes, thereby promoting spatial independence^55^. All landscape metrics (i.e., tree cover, corridor cover, live-fence cover, fragmentation, remnant tree density, patch aggregation index, mean inter-patch distance, largest patch index) were obtained using the FRAGSTATS software^56^.

### Scale of effect

Species-landscape associations depend on the size at which landscape metrics are measured^57^. Therefore, landscape metrics must be measured across different areas to identify the scale of effect (SoE), or the area that yields the strongest species-landscape association^58,59^. We built generalized linear models (GLMs) between each landscape metric and each response variable using the *multifit* function^60^ in R version 3.3.2^61^. We built one GLM for each buffer size, thereby testing 544 models in all (17 buffers × 4 response variables × 8 landscape metrics). All response variables were assessed with a Poisson distribution error, except for bird abundance, as this continuous variable was tested with a Gaussian distribution error^62^. Then we plotted and used the AIC value of each model (as a dependent variable) against landscape size to assess the strength of each relationship (Supplementary Figs. 2 and 3). The selected SoE for all subsequent statistical analyses described below utilized the buffer size at which we observed the lowest AIC value alongside a significant relationship (Supplementary Table 1).

### Multi-model analyses

The first step to decrease the number of landscape metrics in the model selection (Supplementary Fig. 4) was to select non-correlated ecologically-meaningful measures of landscape structure^15,63^. We always kept tree cover as a metric, because we were interested in teasing apart tree cover effects from fragmentation and other configuration metrics effects (i.e., by statistically controlling confounding effects with tree cover). Second, we only retained metrics with a significant effect on response variables. Third, for each full model, we tested for multicollinearity among metrics using the variance inflation factor (VIF) with the *car* package for R. We eliminated metrics with VIF values > 4 from the full model, indicating severe collinearity among metrics^64^.

We then assessed the relative effect of each landscape metric (measured at its scale of effect) on each of the four response variables using an information-theoretic and multi-model averaging approach^65^. For each response variable, we constructed models limiting the number of terms to three landscape metrics using *glmulti* package for R^66^. We calculated the Akaike’s Information Criterion for each model corrected for overdispersion and sample size (_q_AIC_c_) and ranked models from the lowest to highest _q_AIC_c_. We obtained the relative importance for each landscape variable by summing the Akaike’s weight (Σ_wi_) of each model where that variable appeared. This indicates the probability that a given variable is included in the best fitting model^65,67^. Finally, we tested for spatial autocorrelation of our response variables by calculating Moran’s I index with the *ape* package for R^68^ and found very low values (< 0.04) and no spatial autocorrelation (*p* > 0.1).

## Results

We recorded 34 forest-dependent bird species in total. The mean number of species observed within a plot (± SD) was 8.7 ± 4.3 species (range 4–18 species). The mean abundance within plots ranged from 1.1 to 5.8 detections per year. Sample coverage was high in all sites (> 87%), thus suggesting that our sampling effort was adequate, and that our estimates of species richness were not biased by differences in completeness among sites. The total number of late-successional seedlings recorded was 211 individuals (mean = 16.2 seedlings per plot, range = 0–51 seedlings) belonging to 48 species (mean = 7.5 species per plot, range 0–22 species). Only two of the late-successional seedling species recorded were anemochorous whereas the remaining 46 were zoochorous.

At the largest landscape size assessed (i.e., 900-m radius), the mean percentage of tree cover surrounding restoration plots ranged from 30.2 to 79.7%. The percentage of the landscape covered by corridors and live-fences ranged from 10.1 to 62.8% and from 0.8 to 4.8%, respectively. The variation in fragmentation (i.e., forest patch density) was 1.5–9.3 patches per 100 ha, while remnant tree density was 71.2 – 358.0 remnants per 100 ha; the patch aggregation index ranged from 94.9 to 99.0%.

**Figure 2:**
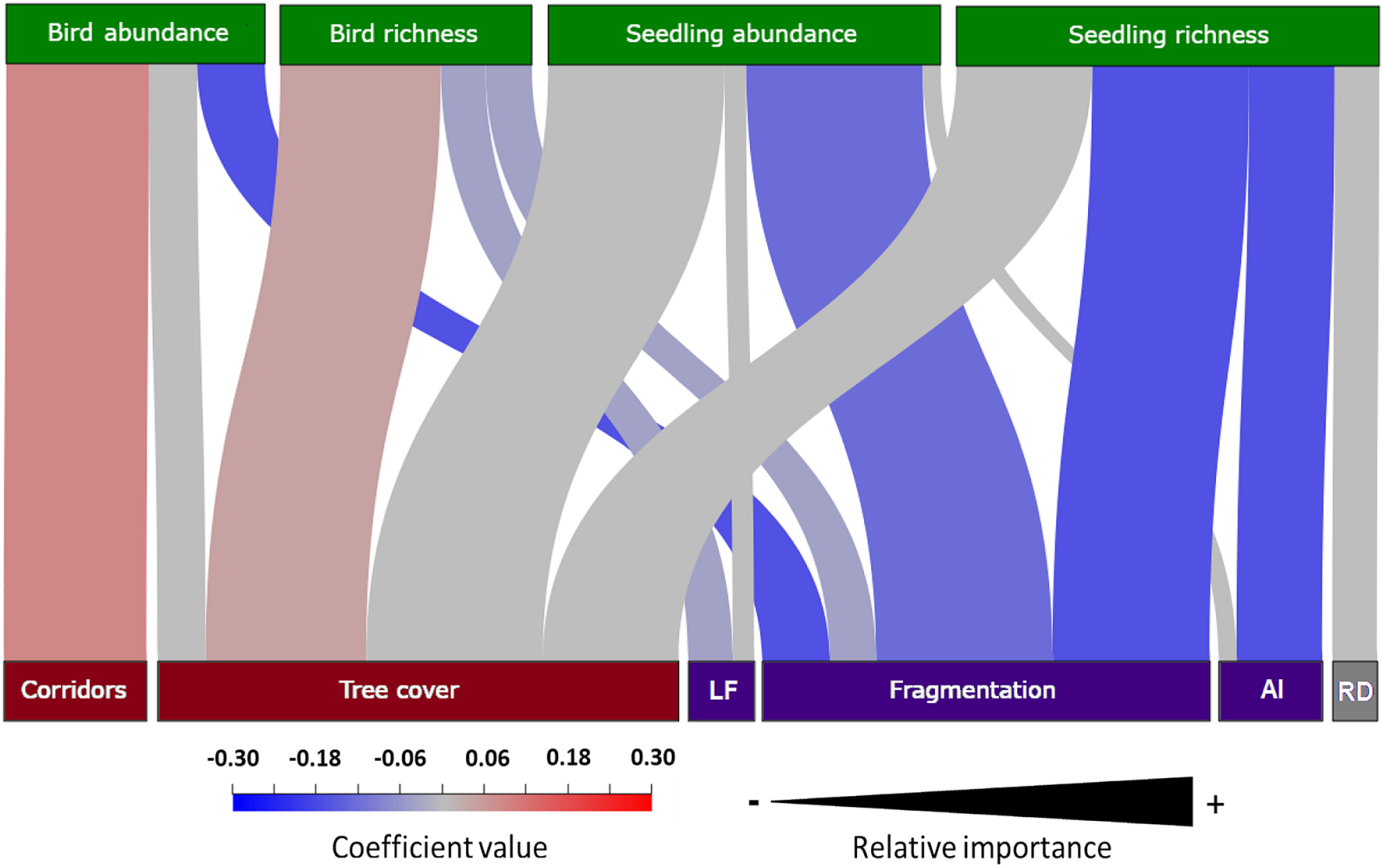
Graphical representation of landscape drivers influencing abundance and richness of highly forest-dependent birds and late-successional tree seedlings in restoration plots in southern Costa Rica. Red boxes indicate landscape metrics with positive effects, blue with negative effects and gray with almost neutral effect. The blue-red gradient represents the coefficient value of the average model that is positive or negative for that factor, respectively. Landscape metrics are the ones included in 95% set of models. The relative importance (Ʃ_wi_) is indicated by the width of the link (0 > Ʃ_wi_ < 1). LF = live-fences, AI = patch aggregation index, RD = remnant tree density.

Multi-model inferences for landscape effects on birds and seedling response variables had the same direction on response variables (Fig. 2). For both birds and seedlings, we found tree cover and fragmentation to be the most important predictors (Fig. 2). Increasing tree cover had positive effects while fragmentation had negative effects. Regarding configuration metrics, fragmentation was more related to seedling than forest dependent bird assemblage response variables, while patch aggregation index and remnant tree density were poor predictors of both taxonomic groups.

### The scale of the effect of landscape structure on birds and seedlings

The scale of landscape effects (SoE) was highly variable among taxa and landscape metrics (Supplementary Figs. 2 and 3; Supplementary Table 1). Mean SoE differed between birds and seedlings (t = 3.44, df = 33.08, *p* = 0.002). Birds had a larger SoE (698 ± 109 m, mean ± SE) than seedlings (312 ± 73 m). Regarding the differences among landscape metrics, overall tree cover, live-fence cover, fragmentation and remnant density showed larger SoE on forest-dependent birds, whereas corridor cover and patch aggregation index showed slightly higher SoE on seedlings (Supplementary Table 1).

### Landscape effects on forest-dependent birds

**Figure 3:**
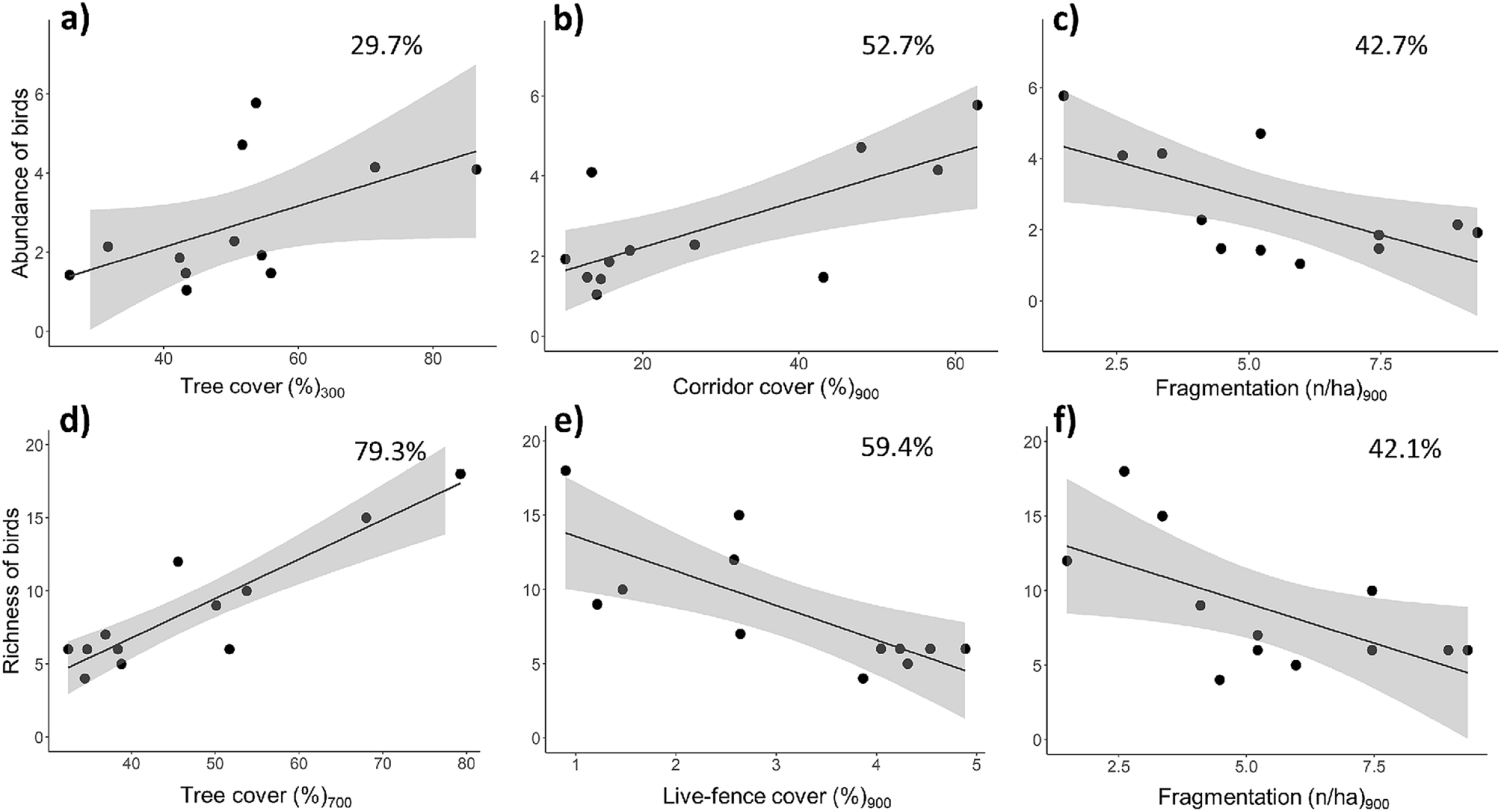
Univariate relationships between the landscape metrics and the abundance and species richness of highly forest-dependent birds in restoration plots in southern Costa Rica. The subscript indicates the scale of effect or landscape radius (m). The black line shows the predicted estimates from the regressions using a generalized linear model. The gray area represents the 95% confidence interval. The percentage of explained deviance (pseudo-R^2^) is shown at the top of the panels. Black points indicate the restoration plots (n = 12).

The abundance of forest-dependent birds was largely explained by corridor and tree cover variables (Table 1; Fig. 2). Bird abundance almost doubled in landscapes with 20% to 60% corridor cover (explained deviance, pseudo-R^2^ = 52.7%; Fig. 3a) and was higher with greater tree cover extent (pseudo-R^2^ = 29.7%; Fig. 3b). Fragmentation had a weaker, negative effect on bird abundance (Figs. 2, 3c). The number of bird species was strongly related to tree cover and live-fences cover, increasing in more forested landscapes (pseudo-R^2^ = 79.3%; Fig. 3d) but decreasing with higher live-fences cover (pseudo-R^2^ = 59.4%). Fragmentation had a relatively weaker effect (Fig. 2), but also decreased bird species richness (Fig. 3e, f). Estimated values and unconditional variances are shown in Supplementary Table 2.

**Table 1:** Best models (i.e., ΔAIC < 2) for assessing the effects of landscape metrics on bird and tree seedling abundance (Ab) and species richness (S) in restoration plots, in southern Costa Rica.

Variable	Best model(s)	AICc	AICc_w_	∆AICc
Highly forest-dependent birds	Ab _Birds_ ~ 1 + CO_900_	43.67	0.37	0
	Ab _Birds_ ~ 1 + CO_900_ + PD_900_	44.79	0.21	1.11
	Ab _Birds_ ~ 1 + TC_300_ + CO_900_	45.05	0.19	1.38
	S _Birds_ ~ 1 + TC_700_	56.86	0.54	0
	S _Birds_ ~ 1 + TC_700_ + FR_900_	58.79	0.20	1.92
Late successional tree seedlings	Ab _Late-successional_ ~ TC_20_ + FR_300_	176.58	0.78	0
	S _Late-successional_ ~ TC_20_ + FR_450_ + AI_600_	91.63	0.40	0
	S _Late-successional_ ~ TC_20_ + FR_450_	92.99	0.20	−1.36

We used qAICc instead of AIC to correct for small sample size (n = 13 for seedlings, n = 12 for birds) and overdispersion. The importance value or AIC weight (AICc_w_) for each model as well as the differences in AIC value (ΔAICc) are given for each model.

*TC* tree cover, *CO* corridor cover, *FR* fragmentation (i.e., forest patch density), *AI* forest patches aggregation index.

### Landscape effects on late-successional recruitment

**Figure 4:**
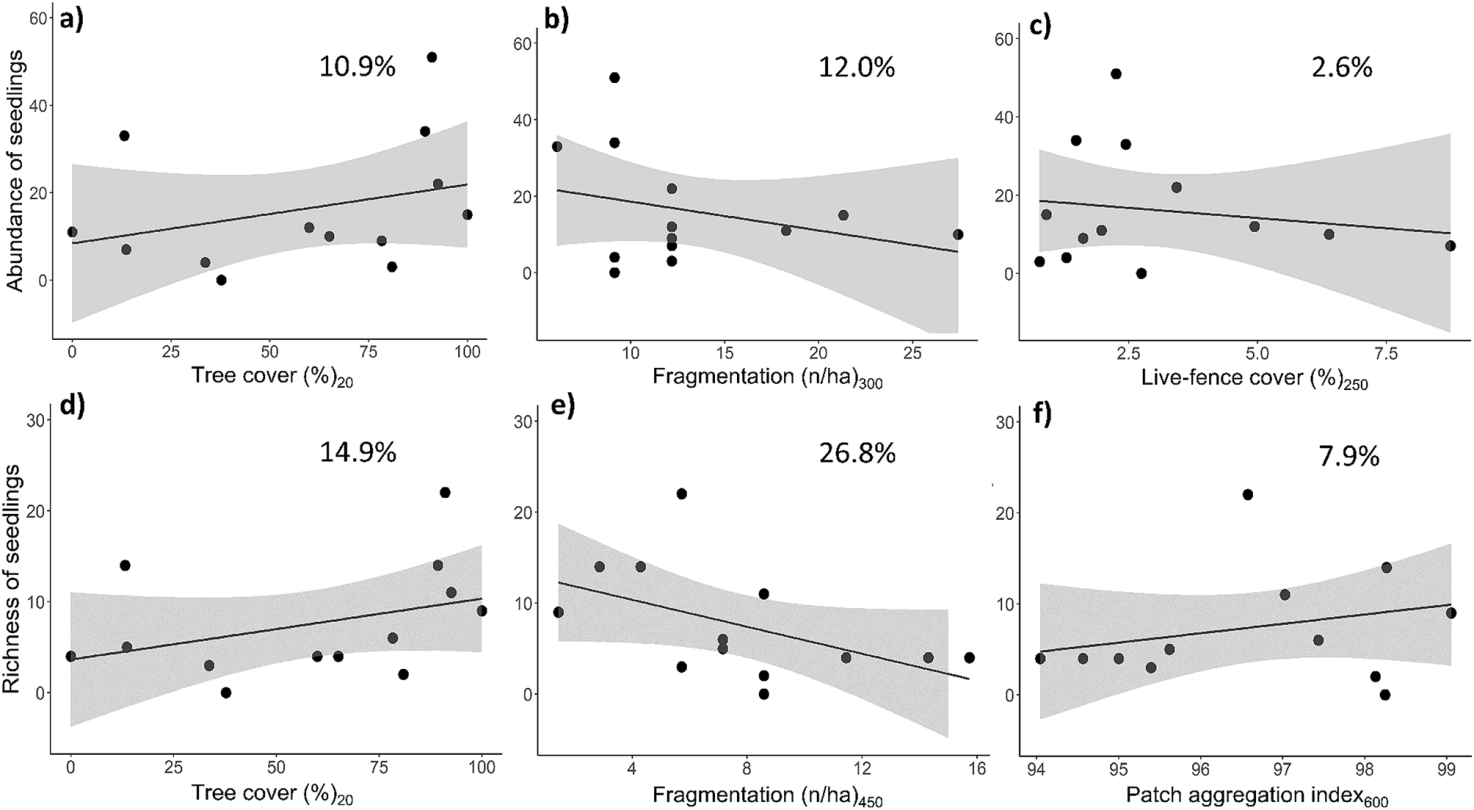
Univariate relationships between the landscape metrics and the abundance and species richness of late-successional seedlings in restoration plots in southern Costa Rica. The subscript indicates the scale of effect or landscape radius (m). The black line shows the predicted estimates from the regressions using a generalized linear model. The gray area represents the 95% confidence interval. The percentage of explained deviance (pseudo-R^2^) is shown at the top of the panels. Black points indicate the restoration plots (n = 13).

The best models for explaining both abundance and species richness of late-successional seedlings included tree cover and fragmentation (Table 1). However, the abundance and the species richness of late-successional seedlings, while similar to responses for forest-dependent birds, were only weakly related to landscape metrics (Fig. 4a–d, f; pseudo-R^2^ < 15.0%) with the exception of fragmentation, which was negatively related to seedling species richness (pseudo-R^2^ = 26.8%; Fig. 4e). In this case, species richness was twice as high in the least fragmented landscape compared to the most fragmented. Estimated values and unconditional variance are noted in Supplementary Table 2.

## Discussion

We use a landscape-scale long-term restoration study to demonstrate that percent tree cover and fragmentation strongly affect visitation by forest-dependent birds and late successional tree seedling recruit abundance and species richness after two decades of forest recovery in restoration plantation plots. As expected, we found that landscape-scale tree cover was important for both bird visitation and seedling recovery, but it had a stronger positive effect on birds, likely because different mechanisms drive their presence and abundance. For example, forest specialist birds prefer and move through forested areas at long distances whereas an individual nearby tree can affect seedling recruitment within the restoration plots.

Birds were highly and positively related to the amount of tree and corridor cover in the landscape. This result supports a previous result using the same study plots, where the compositional similarity of birds to old-growth forests was greatest in restoration plots embedded in landscapes with high surrounding tree cover^33^. Interestingly, below 40% tree cover in 700-m landscapes, bird species richness is halved, supporting the extinction threshold hypothesis^69^ and the concept that it is crucial to preserve at least 40% of tree cover^5^. Also, as expected, birds were more associated with corridors (Fig. 3b), which are elements that significantly improve landscape connectivity^46,70^. This is particularly the case for forest specialists^46^ as it is well known that forest-dependent seed-dispersing birds prefer large forest remnants and corridors to small (< 10 ha) and isolated forest patches, and rarely move through open habitats^54^.

Late successional tree seedlings showed a weaker response to tree cover, possibly because isolated remnant trees can influence recruitment dynamics in restoration plots^23^ and seedlings face strong establishment limitation in addition to dispersal limitation^71^. Another study in the same study region noted that bird abundance was a strong predictor of seed rain richness and abundance, but that landscape structure was a poor predictor^72^. Nonetheless, previous research in the study sites showed that forest cover has a weak effect on the recruitment of the whole tree community^18,20,21^, most likely because many species are rare and tree species in the restoration plots are generally establishment limited^39^, and site-level features strongly drive recruitment^39,73^. For example, seed to seedling transition rates are low in remnant forests, where seed source availability and seed arrival is high^39^.

Contrary to expectations, fragmentation per se (i.e., patch density) had negative effects on both response variables, but particularly on seedlings. When assessing a whole assemblage of species, fragmentation per se mostly has non-significant or if significant then typically it has positive effects, since it increases habitat diversity and promotes landscape complementation and connectivity^25,26^. But here we are looking at forest specialist species, an assemblage that might be more sensitive to edges and fragmentation and require large forested areas to persist while highly fragmented landscapes have more edges and patches are smaller and more aggregated^7^. Furthermore, small forest patches are edge dominated, facilitating human disturbance such as selective logging^74^. Smaller forest patches also tend to select for a particular set of abundant pioneer species^74,75^ and sometimes may lack late-successional adult trees (i.e., seed sources^76^).

Overall, the scale of effect for birds (mean = 700 m) was larger than that found for tree seedlings (mean = 275 m), which is not surprising given that the scale of effect depends on a species’ given dispersal ability among other factors^77^. In this sense, birds are highly mobile and frequently move across the landscape. Many movements occur over long distances^16^. The overall smaller SoE for seedlings indicates that sources of late-successional species might be close to restoration plots. Here the scale of effect of tree cover on seedlings was 20-m and in a prior study in the same restoration plots, trees within a 25 m radius were found to strongly influence recruitment probability within plots^23^. Assessing at which scale biodiversity patterns are responding to the landscape in a given site is very important when designing a restoration project, as this may help to predict which species might need to be planted and which ones will naturally arrive, or even to identify if any planting is needed.

Contrary to expectations, the density of remnant trees and tree cover produced by live-fences were not strong predictors of the abundance and richness of birds or seedlings. This supports a previous study showing that forest-dependent birds tend to avoid moving through an open matrix by means of remnant trees or live-fences^54,70^. For restrictive forest-dependent species, the usage of corridors might be the most important connectivity element within the landscape, as shown in this study and in previous work^46^. The weak association found for forest-dependent birds, some of which drive seed dispersal of forest interior late-successional tree species^72^, might also explain the weak association found between remnant trees and live-fences with seedling recruits as it is forest-dependent birds that would be the main dispersers of late-successional species.

In conclusion, landscape structure effects have the same direction for birds and seedlings, but the strength and scale of the effect varies. Tree cover and fragmentation were the most important variables affecting the abundance and species richness of seedlings and birds. However, seedlings were weakly affected by tree cover in the surrounding landscape, probably because any single nearby tree can act as a seed source (e.g., remnant trees located < 60 m from restoration plot edges, Supplementary Table 1). As such, landscape structure seems to be more associated with birds than it is for seedlings, probably because landscape structure directly affects bird movement across a much bigger distance range, but seedling recruitment depends on disperser movements, the presence of seed sources and post-dispersal establishment limitations. In our region, corridors, and particularly riparian corridors, are essential for the abundance of forest-dependent birds within restoration plots, while other connectivity elements, such as live-fences or remnant trees, seemed to be less important for this particular group. Finally, restoration projects that have particular goals (e.g., recovery of late-successional species), can be better served by an integrated landscape approach. Hence, knowing the extent, and how the landscape may affect restoration outcomes can help to infer what kind of species will arrive to restoration plots. If we aim to implement a network of smaller optimal landscapes^5^ in an ever more resource-limited world, we need to integrate landscape ecology and restoration science to effect better land-use practice.

## Supplementary Information

Supplementary Information.

## Data Availability

The datasets used and/or analyzed during the current study available from the corresponding author on reasonable request.
