# Supplementary material for: Effects of landscape structure on restoration success in tropical premontane forest
Source: Sci Rep. 2022 Aug 4;12:13452. doi: 10.1038/s41598-022-16542-3 (PMC9352795; doi:10.1038/s41598-022-16542-3)
Supplement: Supplementary file 1 — Supplementary Information. [file 41598_2022_16542_MOESM1_ESM.docx]

**Supplementary material**

**Effects of landscape structure on restoration success in tropical premontane forest**

Miriam San-José, Leland K. Werden, Francis H. Joyce, J. Leighton Reid, Karen D. Holl, Rakan A. Zahawi

**Table 1.** Scale of landscape structure effect on the abundance and species richness of forest-dependent birds and late-successional tree seedlings. Non-significant effects are n.s.

| **Response variable** | **Landscape metric** | **Radius (m) with lowest AICc** | **Significance** | **Scale of Effect (SoE)** |
| --- | --- | --- | --- | --- |
| Species richness of forest-dependent birds | | | |  |
|  | Tree cover | 700 |  | 700 |
|  | Live-fences | 900 |  | 900 |
|  | Corridor cover | 450 |  | 450 |
|  | Fragmentation | 900 |  | 900 |
|  | Remnant density | 900 |  | 900 |
|  | LPI | 800 |  | 800 |
|  | ENN | 40 | n.s. | Discarded |
|  | AI | 900 |  | 900 |
| Abundance of forest-dependent birds | | | |  |
|  | Tree cover | 300 | n.s. | 300 |
|  | Live-fences | 500 | n.s. | Discarded |
|  | Corridors cover | 900 |  | 900 |
|  | Fragmentation | 900 |  | 900 |
|  | Remnant density | 700 | n.s. | Discarded |
|  | LPI | 800 | n.s. | Discarded |
|  | ENN | 20 | n.s. | Discarded |
|  | AI | 20 | n.s. | 900 * |
| Abundance of late-successional seedlings | | | |  |
|  | Tree cover | 20 |  | 20 |
|  | Live-fences | 250 |  | 250 |
|  | Corridor cover | 900 |  | 900 |
|  | Fragmentation | 300 |  | 300 |
|  | Remnant density | 60 |  | 60 |
|  | LPI | 20 |  | 20 |
|  | ENN | 20 | n.s. | Discarded |
|  | AI | 20 | n.s. | 600 * |
| Species richness of late-successional seedlings | | | |  |
|  | Tree cover | 20 |  | 20 |
|  | Live-fences | 100 | n.s. | Discarded |
|  | Corridor cover | 900 | n.s. | Discarded |
|  | Fragmentation | 450 |  | 450 |
|  | Remnant density | 60 |  | 60 |
|  | LPI | 20 |  | 20 |
|  | ENN | 20 | n.s. | Discarded |
|  | AI | 20 | n.s. | 600 * |

* We use this landscape radius as ΔAICc with the best model was < 2 and none of the sites had N.A. values.

**Table 2.** Values of model-averaged parameter estimates, unconditional variance and importance for landscape metrics´ effect on the abundance and richness of forest-dependent birds and late-successional seedlings. Ab = abundance, S = number of species, TC= % tree cover in the landscape, CO = % tree cover comprised in corridors in the landscape, FR = fragmentation or patch density (forest polygons >0.25 ha compacted), RD = remnant tree density (forest polygons <0.25 ha compacted), LF = % tree cover comprised of live-fences in the landscape, AI = patch aggregation index. Subindex refers to the scale of effect or radius size at which relationship between variables were stronger.

| **Complete model** | | **Estimate** | **Unconditional variance** | **Importance value** | **alpha value** |
| --- | --- | --- | --- | --- | --- |
| Ab _Birds_ ~ TC_300_ + FR_900_ + CO_900_ | | |  |  |  |
|  | CO_900_ | 0.041 | 0.001 | 0.796 | 0.053 |
|  | FR_900_ | -0.115 | 0.032 | 0.379 | 0.331 |
|  | TC_300_ | 0.01 | 0.000 | 0.270 | 0.033 |
|  | (Intercept) | 1.68 | 3.660 | 1.000 | 3.534 |
|  |  |  |  |  |  |
| S _Birds_ ~ TC_700_ + FR_900_ + LF_900_ | | |  |  |  |
|  | TC_700_ | 0.022 | 0.000 | 0.902 | 0.019 |
|  | FR_900_ | -0.017 | 0.001 | 0.264 | 0.062 |
|  | LF_900_ | -0.036 | 0.008 | 0.248 | 0.134 |
|  | (Intercept) | 1.268 | 0.582 | 1.000 | 1.432 |
|  |  |  |  |  |  |
| Ab _Seedlings_ ~ TC_20_ + LF_250_ + FR_300_ + AI_600_ | | | | | |
|  | TC_20_ | 0.011 | <0.001 | 0.999 | 0.005 |
|  | FR_300_ | -0.073 | <0.001 | 0.999 | 0.037 |
|  | LF_250_ | 0.004 | <0.001 | 0.122 | 0.020 |
|  | AI_600_ | -0.002 | <0.001 | 0.095 | 0.015 |
|  | (Intercept) | 3.134 | 2.737 | 1 | 1.691 |
| S _Seedlings_ ~ TC_20_ + RD_60_ + FR_450_ + AI_600_ | | | |  |  |
|  | FR_450_ | -0.117 | 0.004 | 0.879 | 0.128 |
|  | TC_20_ | 0.007 | <0.001 | 0.760 | 0.010 |
|  | AI_600_ | -0.120 | 0.023 | 0.484 | 0.302 |
|  | RD_60_ | -0.001 | <0.001 | 0.247 | 0.003 |
|  | (Intercept) | 14.148 | 225.274 | 1 | 29.626 |


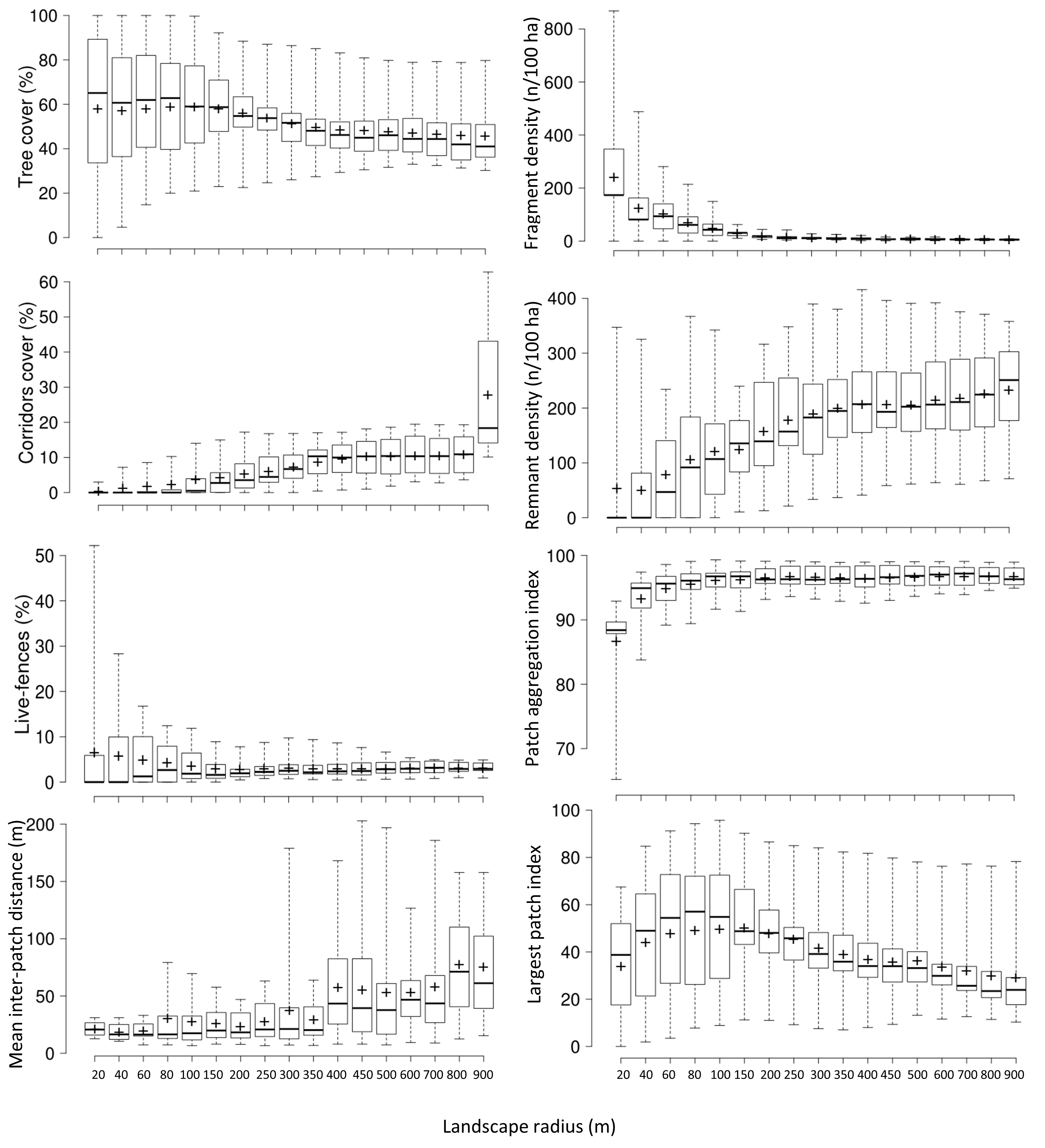
 **Figure 1.** Landscape metrics measured at different landscape radii (range = 20 – 900 m) around plantation restoration plots in southern Costa Rica. Center lines show the medians; box limits indicate the 25th and 75th percentiles; whiskers extend to minimum and maximum values; crosses represent sample means. n = 13 landscape units


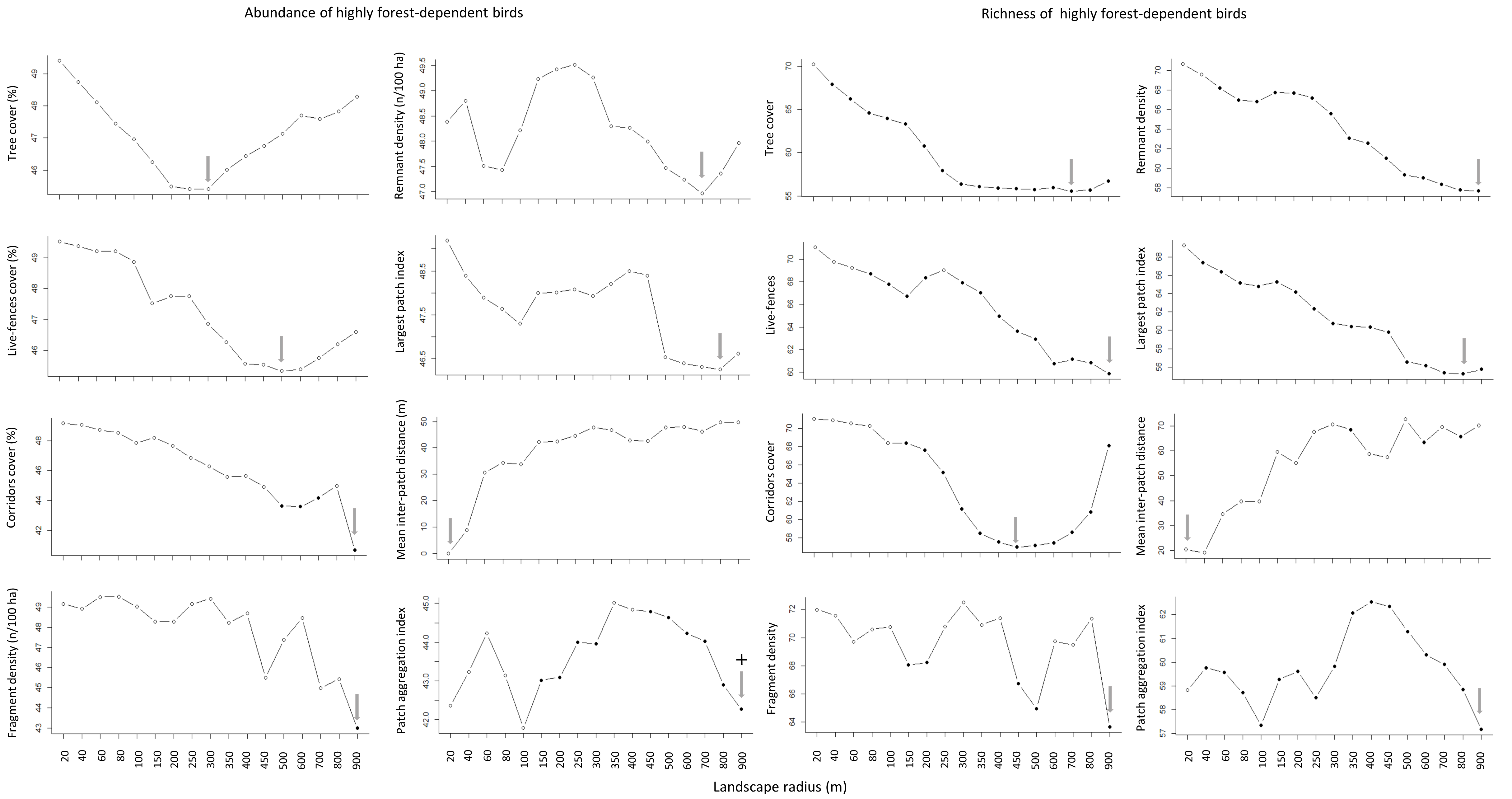
 **Figure 2.** Scale of effect (SoE) of landscape metrics on the abundance and species richness of forest-dependent birds in restoration plots in Southern Costa Rica. The y-axis shows the AIC value for the models of each landscape metric at landscape radius. Black dots indicate significative effects and white dots non-significative effects. + indicates a case where the SoE was the smallest radius but there were many NA values, hence, we selected the next best model with a difference in AIC value <2.


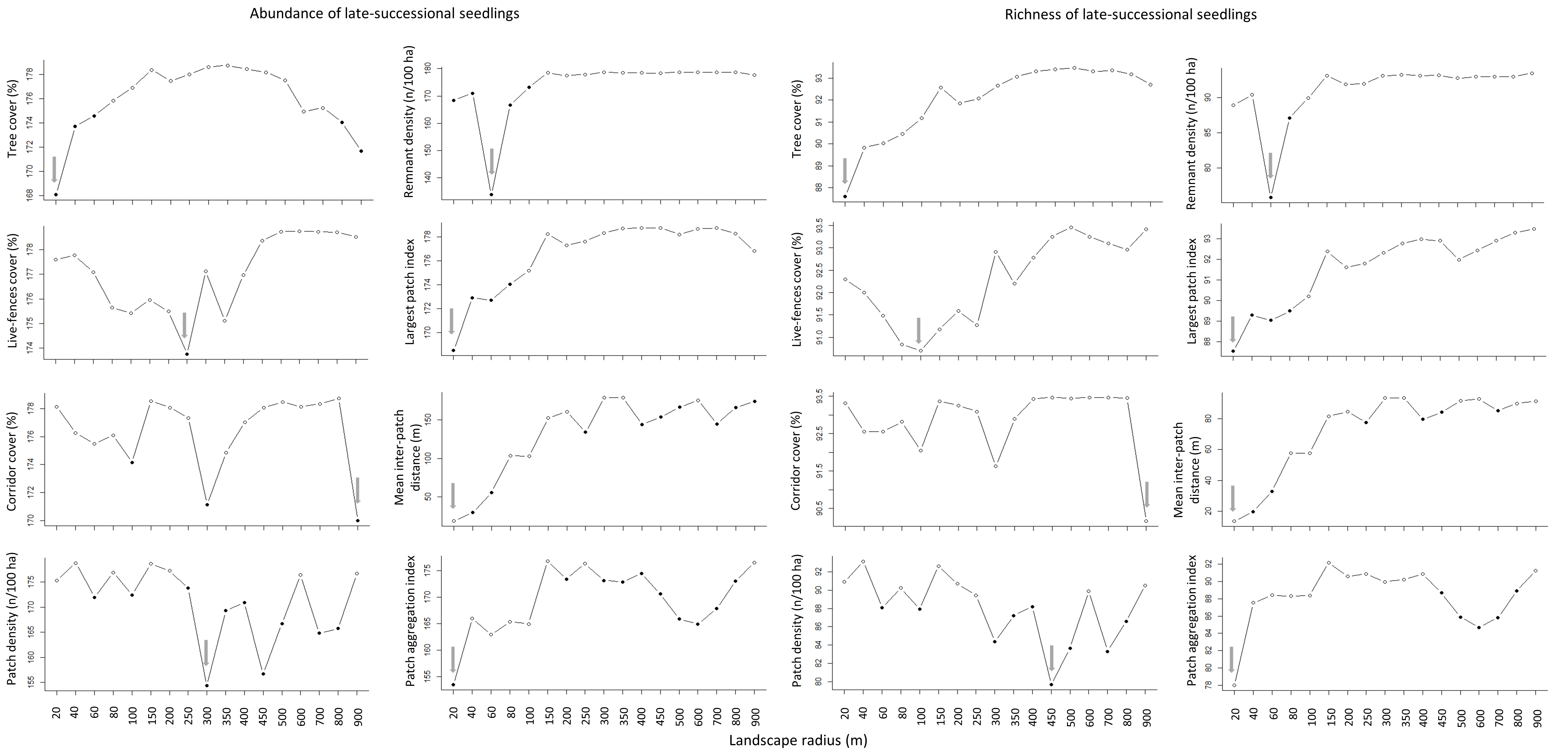
 **Figure 3.** Scale of effect of landscape metrics on the abundance and species richness of late-successional tree seedlings in restoration plots in southern Costa Rica. The y-axis shows the AIC value for the models of each landscape metric at landscape radius. Black dots indicate significative effects and white dots non-significative effects.


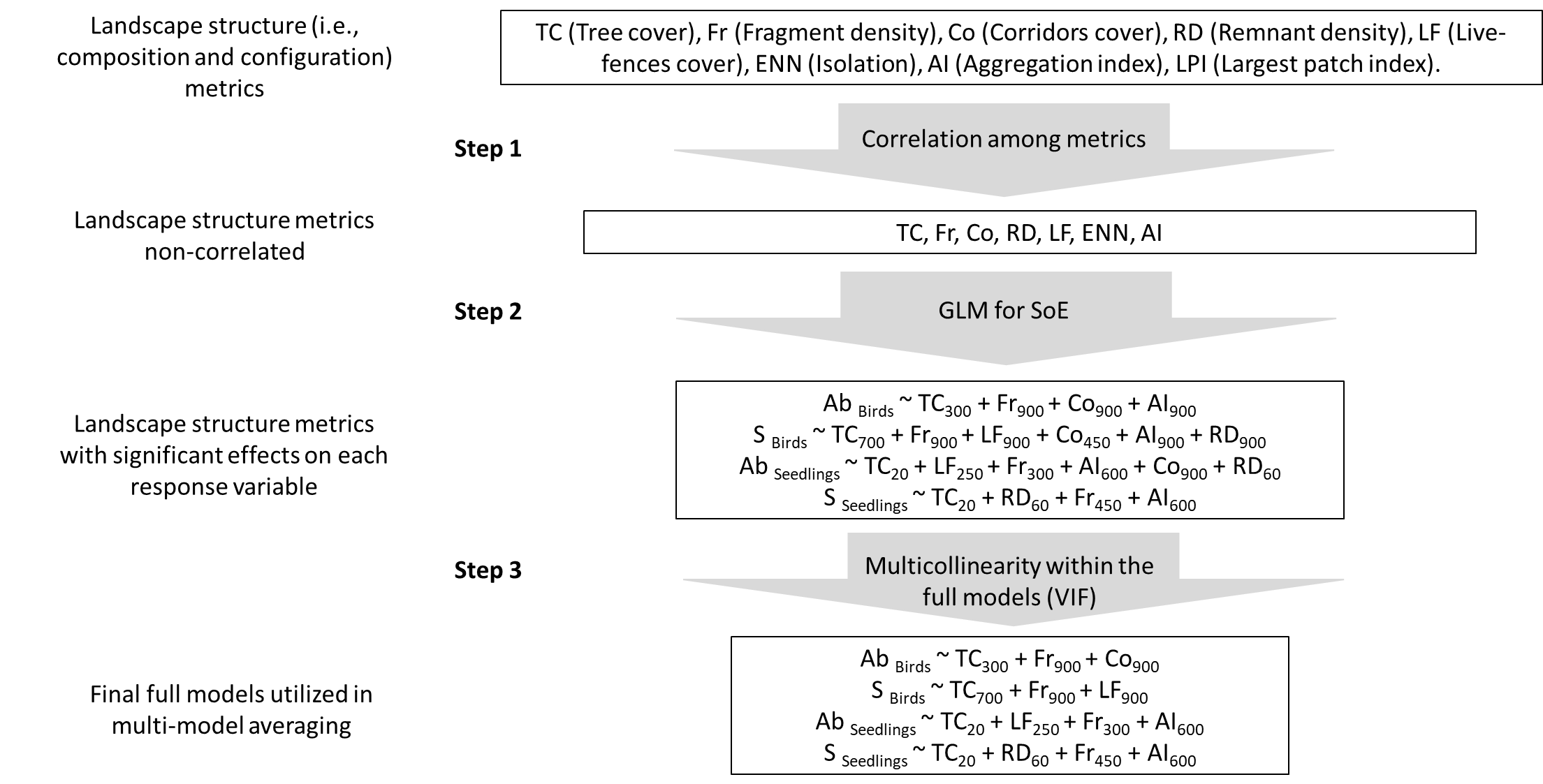


**Figure 4.** Flow chart showing the methodology followed to select landscape predictors for multi-model averaging inference.
